# Adjuvant treatment of chronic osteomyelitis of the tibia following exogenous trauma using OSTEOSET^®^-T: a review of 21 patients in a regional trauma centre

**DOI:** 10.1007/s11751-014-0206-y

**Published:** 2014-12-25

**Authors:** Gemma Humm, Saqib Noor, Philippa Bridgeman, Michael David, Deepa Bose

**Affiliations:** 1 Queen Elizabeth Hospital, University Hospitals Birmingham NHS Foundation Trust, Mindelsohn Way, Birmingham, B15 2GW UK; 2 East and North Hertfordshire NHS Trust, Lister Hospital, Coreys Mill Lane, Stevenage, SG1 4AB UK

**Keywords:** Osteomyelitis, Tibia, Trauma, Osteoset, Antibiotic

## Abstract

Surgical debridement and prolonged systemic antibiotic therapy are an established management strategy for infection after tibial fractures. Local antibiotic delivery via cement beads has shown improved outcome but requires further surgery for extraction of beads. OSTEOSET^®^-T is a resorbable bone void filler composed of calcium sulphate and 4 % tobramycin that is packed easily into bone defects. This is a review of the outcomes of 21 patients treated with OSTEOSET^®^-T for osteomyelitis of the tibia. This is a retrospective case note and clinical review. In all cases, the strategy was debridement, with removal of any implants, with excision back to bleeding bone. OSTEOSET^®^-T pellets were packed into any contained defects or the intra-medullary canal with further bony stabilisation (*n* = 9) and soft tissue reconstruction (*n* = 7) undertaken as required. Intravenous vancomycin and meropenem were administered after sampling with substitution to targeted antibiotic therapy for between 6 weeks and 6 months. The average follow-up was 15 months. Union rate after tibial reconstruction was 100 %. Wound complications were encountered in 52 %: a wound discharge in the early post-operative period was noted in seven patients (33 %) independent of site of pellet placement. In the 14 cases without a wound leak, five developed wound complications (*p* = 0.06, Fisher’s exact test) either from delayed wound-healing or pin-site infections. One patient developed a transient acute kidney injury and one refractory osteomyelitis. OSTEOSET^®^-T is an effective adjunct in the treatment of chronic tibial osteomyelitis following trauma based on the low incidence of relapse of infection within the period of follow-up in this study, but significant wound complications and one transient nephrotoxic event were also recorded.

## Introduction

Antibiotics are an important part of the strategy in treating infection after tibial fractures. In established osteomyelitis, surgical debridement is followed with systemic and, sometimes, local antibiotic delivery. Antibiotic-impregnated cement beads are frequently used as an adjunct to delivery of antibiotics but often require a further surgical procedure for removal.

The ability to treat chronic osteomyelitis with single-stage surgery potentially reduces the risk and morbidity associated with repeated operative procedures and general anaesthetic. Reduction in theatre time and length of in-patient stay are added economic benefits. Despite single-stage surgery showing reasonable success in achieving union in infected non-union of long bones, persistent infection and subsequent revision surgery may be needed [1].

Calcium sulphate has been used successfully in the treatment of non-union and is an osteo-conductive void filler that is resorbed at a rate similar to that of bone formation [2, 3]. OSTEOSET^®^-T (Wright Medical Technology Inc. Arlington TN USA) comes pre-packaged in small pellets to allow easy packing into bone. It comes preloaded with 4 % tobramycin, and drug elution profiles have shown levels up to 10,000 times the minimum inhibitory concentration for most strains of *Staphylococcus* [4]. No further surgery is required to remove the resorbable pellets. Animal studies have demonstrated that its use prevents intramedullary and post-operative wound infection following the treatment of open, contaminated long-bone fractures [5, 6].

OSTEOSET^®^-T has been used as a bone graft substitute with success in the management of infected non-union of the tibia. Management involves radical debridement of infected bone and placement of gentamicin-impregnated beads, prior to definitive fixation and the use of OSTEOSET^®^-T [7]. Union was achieved without the need for autologous bone graft and without recurrence of infection. A retrospective comparison of the use of OSTEOSET^®^-T with debridement versus debridement alone has supported the use of OSTEOSET^®^-T in single-stage surgery in the treatment of adult osteomyelitis. Chang et al. [8] have described the use of vancomycin with OSTEOSET^®^-T for cases of tobramycin resistance. However, it has been associated with wound complications, e.g. in the development of sinuses draining a sterile effluent, but this was found to be self-limiting and resolved after the complete absorption of OSTEOSET^®^-T and without recurrence of infection [9, 10].

The aminoglycosides used in the treatment of bone infections belong to the protein synthesis inhibitor family of antibiotics, binding to the bacterial ribosomal 30S subunit to achieve a bacteriostatic effect through transcription errors during cell division [11]. An additional ability to disrupt bacterial cell membranes accounts for its bactericidal properties [12]. They have a broad spectrum and are effective against both gram-positive and gram-negative organisms [13]. *Pseudomonas aeruginosa*, *Staphylococcusaureus* and *Enterobacteriaceae* are sensitive to tobramycin which has a lower side-effect profile than gentamicin [14].

This case series reports on the outcome of 21 patients with chronic osteomyelitis managed by single-stage surgery and using OSTEOSET^®^-T as an adjunct to intravenous antimicrobial therapy.

## Materials and Methods

Twenty-one cases of chronic tibial osteomyelitis in which treatment involved the use of OSTEOSET^®^-T as a space filler and local antibiotic delivery system were identified by a retrospective review over a 30-month period from 2010 to 2012. Data were collected using a proforma and included demographics, record of the intra-operative procedure, relevant microbiology, renal function, complications relating to recurrent infection and wound healing, the presence of a wound leak and repeat surgeries. A wound leak was defined subjectively as a serous leakage considered a potential risk to normal wound healing [15]. A standardised surgical protocol and antibiotic regime based on guidance from our local microbiology department were followed.

In all cases, surgery was directed at excising infected tissue and sinuses; a radical debridement was performed, guided by pre-operative magnetic resonance imaging, until bleeding confirmed on residual bone. Metalwork, if present, was removed. Once complete, OSTEOSET^®^-T pellets were packed into any defects or into the intra-medullary canal in those cases where an intramedullary nail had been removed. Further, tibial stabilisation and soft tissue cover were carried out as deemed necessary. The protocol involved taking a minimum of five tissue samples from deep tissues using fresh instruments for each sample. Empiric intravenous vancomycin and meropenem were administered after samples were taken. Meropenem was discontinued after 3 days in the absence of gram-negative growth, and vancomycin continued until the 7-day culture results became available. Thereafter, targeted antibiotic therapy was continued for a period of 6 weeks to 6 months, or empiric ciprofloxacin and rifampicin if no growth was seen. This approach is based on the probability of involvement of a *Staphylococcus* bacterium species contraposed to local antibiotic resistance patterns and the previous published literature on the effectiveness of combination of dual therapy [15–17].

Data analysis and parametric tests were performed using Microsoft Excel for Mac 2011, while nonparametric statistical tests done with GraphPad QuickCalcs (2013 GraphPad Software, Inc.). Statistical significance was set at *p* value of ≤0.05.

## Results

**Fig. 1:**
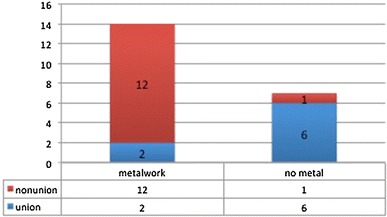
Cases of non-union and the presence of metal work

There were 18 males and 3 females with a mean age of 49 (range 26–88) and follow-up of 16 months (range 6–25). Six patients were classified as Cierney-Mader grade 3A, one patient as grade 3B, 13 patients grade 4A, and one patient grade 4B. In situ metal work was removed (Fig. 1). Nine cases required fixation: eight with circular external fixators and one with a monolateral external fixator. Seven cases required reconstructive soft tissue cover provided by our resident plastic surgical team: there were four local flaps and three free vascularised flaps.

Microbiological analysis of the samples revealed 38 % of the infections were caused by a strain of *Staphylococcus* species. A significant number either had mixed growth or no identified growth. This is summarised in Table 1. Renal function was assessed in all cases. One case was complicated by a transient post-operative acute kidney injury which resolved after 1 week without the need for renal support. This patient had pre-existing comorbidities of obesity and essential hypertension.

**Table 1:** Causative organisms involved

Organism	Cases	%
Polymicrobial	4	19
Coagulase-negative *Staphylococci*	4	19
*Staphylococcus aureus*	4	19
Negative cultures	3	14
Other organisms	6	29
*Serratia* sp.	2	
*Corynebacterium* sp.	1	
*Enterococcus* sp.	1	
Mixed anaerobes	1	
*Propionibacterium*	1	

**Fig. 2:**
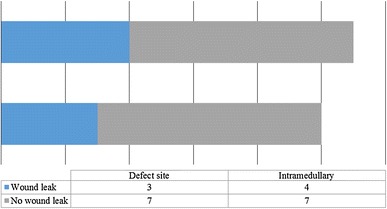
Impact of site of placement of OSTEOSET^®^-T placement and the presence of a post-operative wound leak (*p* = 1.0, Fisher’s exact test)

**Fig. 3:**
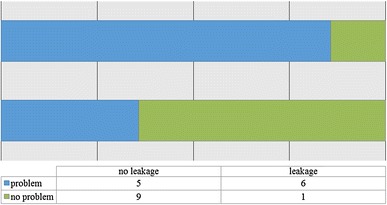
Impact of wound leakage on healing (*p* = 0.06, Fisher’s exact test)

Seven cases were complicated by wound leakage; the location of OSTEOSET^®^-T pellets in either a closed intramedullary cavity or an open cortical defect did not relate to its incidence (Fig. 2). Three-quarters of these cases with a wound leak went on to have problems with wound healing as compared to just one-third of the cases without a leak. A serous leak appears to double the relative risk of wound-healing problems (Fig. 3).

Successful eradication of infection, as determined in the time of follow-up in this series, was achieved in 20 cases. Only one patient required further debridement and soft tissue coverage and remained free of continuing infection at latest follow-up. One patient required further surgery for the correction of residual deformity.

**Fig. 4:**
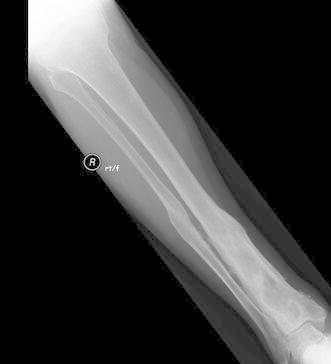
Pre-operative plain radiograph of the right distal tibal in a 57 year old male with chronic post-traumatic osteomyelitis distal tibia

**Fig. 5:**
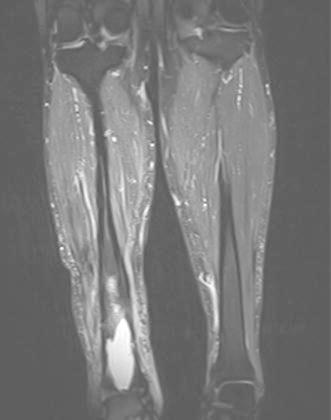
MRI appearances of chronic osteomyelitis of the right distal tibia

**Fig. 6:**
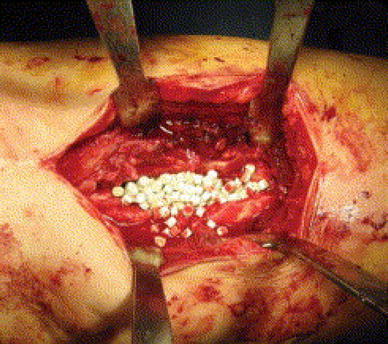
Intra-operative use of OSTEOSET^®^-T

**Fig. 7:**
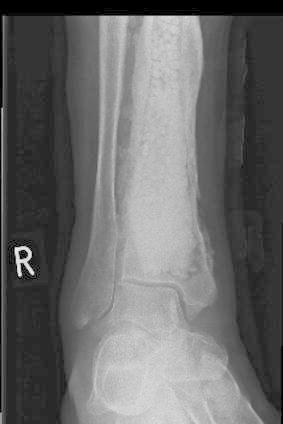
Post-operative plain radiograph of right distal tibia packed with OSTEOSET^®^-T

**Fig. 8:**
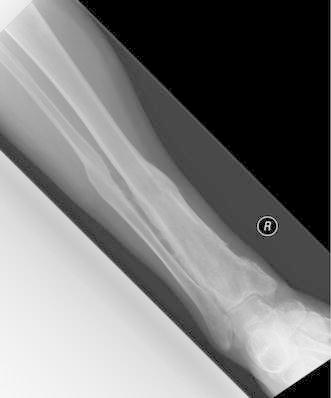
Plain radiograph of the right distal tibia following second stage bone grafting

Figures 4, 5, 6, 7 and 8 show the successful management of a 57 year old man with post traumatic chronic tibial osteomyelitis using OSTEOSET^®^-T as an adjunct to surgical debridement.

## Discussion

This case series included a sample of patients with chronic osteomyelitis of the tibia secondary to trauma. The use of OSTEOSET^®^-T was an effective adjunct with eradication of infection in all of our cases in the period of follow-up, although one patient required a further surgical debridement owing to an unhealthy appearance of the wound and suspected persistent infection. The occurrence of a sterile effluent was a significant complication, as was its associated risk of impaired wound healing. Reported concerns over acute kidney injury (potential nephrotoxic side-effect of high local concentrations of aminoglycosides) were unfounded in our series; the one incident of transient acute kidney injury seen was thought to have occurred from the intravenous antimicrobial therapy in a patient with a background of hypertensive nephropathy.

This case series is limited by the following: the absence of a control group, small numbers and the minimum follow-up period of 6 months. A retrospective case–control study, which contained a mixed group of long-bone infections, found favour in the use of OSTEOSET^®^-T [8]. Prospective data on OSTEOSET^®^-T are limited, with published work concentrating on post-traumatic osteomyelitis in long bones but without comparison with controls [9].

## Conclusion

This case series supports continued use of OSTEOSET^®^-T as an *adjunct* in the treatment of chronic osteomyelitis of the tibia following trauma but highlights potential issues with wound problems. Further prospective and controlled studies are needed to evaluate the role of local antibiotic delivery systems in the treatment of chronic osteomyelitis.
